# Analyzing the Reasons and Hospital Admission Rates of 72‐Hour Emergency Department Revisits

**DOI:** 10.1155/emmi/5425429

**Published:** 2025-12-29

**Authors:** Abdulaziz Alalshaikh, Bader Alyahya, Badr Aldawood, Abdulaziz S. Almehlisi, Sara Almubrik, Sarah Alaidarous, Abdulrahman Alrajhi, Abdulaziz Alhussainy, Mohammed Alageel

**Affiliations:** ^1^ Emergency Medicine Department, King Saud University, College of Medicine, Riyadh, Saudi Arabia, ksu.edu.sa; ^2^ Emergency Medicine Department, King Abdulaziz Medical City, National Guard Health Affairs, King Saud bin Abdulaziz University for Health Sciences, College of Medicine, Riyadh, Saudi Arabia, ksau-hs.edu.sa; ^3^ Emergency Medicine Department, The University of British Columbia, Vancouver, British Columbia, Canada, ubc.ca

**Keywords:** discharge failure, emergency medicine revisits, emergency medicine visits, return visits

## Abstract

**Introduction:**

Emergency department (ED) revisits are considered a significant indicator of the quality of care provided and are used as a benchmark for the performance of individual providers and institutions. The aim of this study is to assess ED revisit rates, reasons, and hospital admission rates among our adult ED patients.

**Methods:**

This is a retrospective chart review study conducted in a single‐center tertiary referral hospital in Riyadh, Saudi Arabia. Study participants comprised adult patients who attended the ED, had been discharged, and had an ED revisit within 72 h from April 2019 to January 2020.

**Results:**

A total of 573 patients met our inclusion criteria, of whom 53.1% were males. The majority of the patients (74.5%) revisiting the ED were categorized as CTAS Level 3, with gastrointestinal complaints being the most common presentation for revisits (23.6%). During the second visit, 94%, 4%, 0.7%, and 0.3% of the participants were discharged, admitted, discharged against medical advice, and died, respectively. Disease progression was the most common cause of revisits at 96.5%. The factors that showed statistically significant associations with nondischarge disposition in the second visit were CTAS levels in the first and second visits, dementia, functional dependency, and reason for the revisit.

**Conclusion:**

Most ED visits within 72 h are due to disease progression rather than system‐ or physician‐related issues, and the majority of these patients are safely discharged after the second visit. Identification of high‐risk patients—such as those with higher CTAS levels, dementia, or functional dependency—may aid emergency physicians in implementing targeted discharge planning and coordinated outpatient follow‐up to reduce unnecessary revisits and optimize use of emergency services. Our findings highlight the importance of structured post–discharge support and underscore the need for tailored interventions in resource‐limited healthcare settings.

## 1. Introduction

Emergency department (ED) revisits are considered a significant indicator of the quality of care provided and are used as a benchmark for the performance of individual providers and institutions, requiring regular review and audit [1–3]. These revisits have identifiable causes in the literature, with several of them being modifiable, including failure to reach the correct diagnosis, improper management, premature discharge, inadequate follow‐up provided, insufficient discharge instructions, and patient noncompliance. These causes are frequently grouped into specific factors, including disease factors, patient factors, physician factors, system factors, or a combination of them [1, 2, 4, 5].

An unscheduled ED revisit is most frequently defined as a patient presenting with the same problem within a specific time frame from discharge, with 72 h being the most commonly investigated cutoff [6, 7]. Alternatively, some studies have defined a revisit as occurring within 48 h of discharge [8]. Previous literature had demonstrated that the rates of these revisits ranged between 0.4% and 15.8%, with a mean of 4.3% [4, 9].

Unscheduled ED revisits have several negative impacts on the patients and healthcare system, thereby leading to ED overcrowding, prolonged patient waiting time, increased healthcare service costs, and increased risk of medicolegal consequences. Moreover, it has been shown to negatively influence the performance and job satisfaction of ED physicians and ancillary staff [5–7, 10, 11].

Pierce JM et al. reported that patients who revisit the ED have a twofold increased admission rate [9]. Several studies have shown that the hospital admission rates of this group vary from 21% to 48%, with a mean of 32.3% [2–13]. Moreover, when admitted, these patients carry higher in‐hospital complication rates reaching 21.7%, including unscheduled surgery, intensive care unit admission, suffering acute cardiovascular conditions, and an increased in‐hospital mortality rate of 7.1% [14].

Internationally, several studies have been conducted to assess ED revisits, focusing on causes and hospital admission rates [4, 5, 9, 13–16]. In Saudi Arabia, two studies have been conducted to assess the rates and reasons for ED revisits among adult and pediatric patients. However, both studies were selective of patients with chronic diseases and from a single institution [17, 18]. To the best of our knowledge, no studies assessing ED revisit rates, reasons, and hospital admission rates among the general adult ED patient population in Saudi Arabia have been conducted.

## 2. Methods

### 2.1. Study Design and Population

This is a retrospective chart review study conducted in a single‐center tertiary referral hospital in Riyadh, Saudi Arabia.

It is a government‐funded hospital serving university faculty and their dependents as well as a selected population from the community requiring tertiary care services. It has an annual census of approximately 165,000 ED visits.

Our ED caters to adult and pediatric patients (≥ 14 years old); it has a separate obstetrics/gynecology area and does not have onsite ophthalmology services. Study participants comprised adult patients who attended the ED, had been discharged, and had an ED revisit within 72 h from April 2019 to January 2020. Patients were included if the second ED visit was deemed related to the initial visit. Data of the participants were collected from the hospital electronic medical record system (E‐SiHi).

Patients’ variables gathered included demographics, Canadian Triage and Acuity Scale (CTAS) levels, primary complaints (categorized into 16 different subcategories), comorbidities, level of dependency, whether the patients were new to the institution, had a history of psychiatric illness, shift time during visits, and final disposition of patients during the second visit (discharge, leaving against medical advice, admission to floor/intensive care unit, and death during the second visit).

Revisit data were obtained from the ED quality department, which regularly collects and audits revisit data using a 72‐h cutoff. For the primary outcome, the “cause of revisit” of the participants who had their second visit was reviewed by the study coauthors and was subsequently categorized according to the reason for the revisit as disease progression, which was defined as a revisit owing to the presenting condition’s worsening or failure of improvement of symptoms, physician‐related, patient‐related, adverse medication, medical intervention effect, and system‐related. For participants whose “cause of revisit” (20 patients) cannot be determined by the study coauthors, two independent physicians adjudicated their categorization.

The following were the exclusion criteria: patients observed in the obstetrics/gynecology section of the ED; patients who left the ED without being examined during the first or second visits; patients who left the ED against medical advice during the initial visit; patients who experienced an acute sickle cell pain crisis; and patients who planned revisits through the ED for direct admission, assessment, or treatment.

### 2.2. Ethical Consideration and Data Analysis

The Institutional Review Board (IRB) of the hospital approved this study (IRB No.: E‐21‐6012). This study was conducted in accordance with the Helsinki Declaration and Good Clinical Practice guidelines. Patient consent was not required owing to the observational nature of this study, and no patient identifiers were collected.

Data were collected using Excel, and statistical analysis was performed using Statistical Package for the Social Sciences version 25.0 for Windows.

## 3. Results

**Figure 1:**
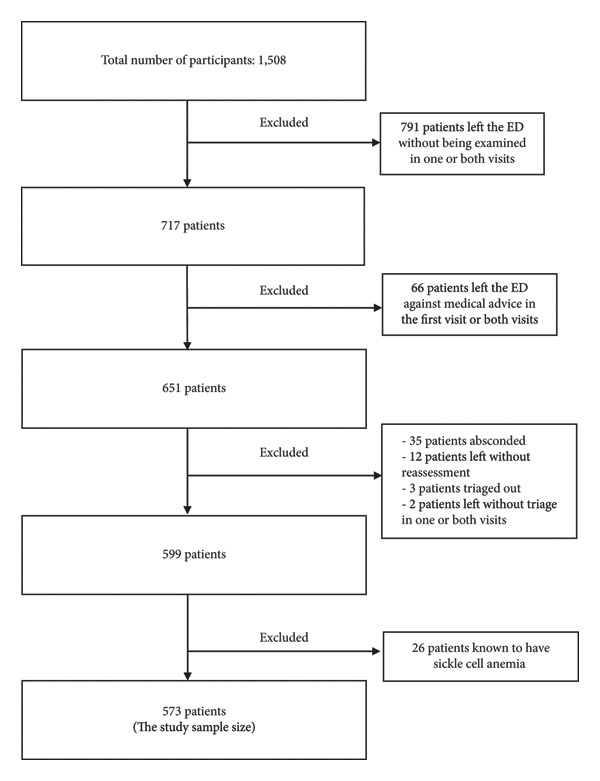
Flowchart of patient exclusion criteria.

The flowchart summarizes patient selection and exclusion criteria for the study cohort. As shown in Figure 1, of 1508 patients screened, 791 were excluded due to leaving the ED without being examined on one or both visits, and additional exclusions were made for absconding, lack of reassessment, or leaving against medical advice. Ultimately, 573 patients formed the final analytic sample for further assessment of early ED revisits.

As shown in Table 1, this study included a total of 573 patients, of whom 53.1% were males. The majority of the patients (74.5%) revisiting the ED were categorized as CTAS Level 3, with gastrointestinal complaints being the most common presentation for revisits (23.6%). Most of the participants (99%) had no formal diagnosis of dementia, and very few had functional dependency (2.1%). Approximately 36% of the participants in the first and second visits presented during the evening shift. Only 17.5% of the patients were new to our institution. During the second visit, 94%, 4%, 0.7%, and 0.3% of the participants were discharged, admitted, discharged against medical advice, and died, respectively. Upon chart review, disease progression was the most common cause of revisits at 96.5%, with only one revisit due to physician care.

**Table 1:** Patients’ characteristics.

Characteristics	N	%
Age (mean, SD)	40.04	16.32
Sex		
Male	304	53.1
Female	269	46.9
Comorbidities		
HTN	100	17.5
DM	87	15.2
Asthma	70	12.2
Dyslipidemia	45	7.9
Autoimmune disease	20	3.5
CAD	11	1.9
CKD	10	1.7
HF	7	1.2
Cancer	7	1.2
Immunodeficiency	0	0.0
Other	139	24.3
Psychiatric diseases		
Depression	13	2.3
Anxiety	13	2.3
CTAS level in the first visit		
1	1	0.2
2	27	4.7
3	303	52.9
4	223	38.9
5	19	3.3
CTAS level in the second visit		
1	2	0.3
2	20	3.5
3	427	74.5
4	118	20.6
5	6	1.0
Diagnosis categories		
GIT	135	23.6
Infectious	104	18.2
Urology–nephrology	61	10.6
CVS	47	8.2
MSK	47	8.2
Pulmonary	43	7.5
Neurology	38	6.6
Trauma	26	4.5
ENT	8	1.4
Vascular	7	1.2
Hematology	7	1.2
Gynecology	6	1
Autoimmune	5	0.9
Oncology	2	0.3
Ophthalmology	1	0.2
Other	36	6.3
Dementia		
No	567	99.0
Yes	5	0.9
Functional dependency		
Dependent	11	1.9
Independent	561	97.9
Shift period (first visit)		
Morning	193	33.7
Evening	210	36.6
Night	170	29.7
Shift period (second visit)		
Morning	218	38.0
Evening	206	36.0
Night	149	26.0
New to the institution		
No	473	82.5
Yes	100	17.5
Disposition (second visit)		
Admission	23	4.0
Discharge	543	94.8
Discharge against medical advice	4	0.7
Death	2	0.3
Place of admission		
Ward	22	3.8
ICU	1	0.2
Not applicable	550	96.0
Death in the second visit or following admission in the second visit		
No	569	99.3
Yes	3	0.5
Reason for the revisit		
Disease progression	553	96.5
Adverse medication and medical intervention effects	16	2.8
System‐related	3	0.5
Physician‐related	1	0.2

Chi‐square and exact tests were used for investigating the factors associated with disposition in the second visit (Table 2). The factors that showed statistically significant associations with nondischarge disposition in the second visit were CTAS levels in the first and second visits, dementia, functional dependency, and reason for the revisit.

**Table 2:** Factors associated with discharge versus nondischarge disposition in the second visit.

			Disposition in the second visit		*P*‐value
			Admission, DAMA, or death (*N* = 29)	Discharge (*N* = 543)	
Age			49.21 (22.10)	39.55 (15.84)	0.027
Sex	Male	N	15	289	0.875
		%	4.90%	95.10%	
	Female	N	14	254	
		%	5.20%	94.80%	
CTAS level in the first visit	1 or 2	N	4	23	**0.001**
		%	14.8%	85.2%	
	3	N	21	282	
		%	6.9%	93.1%	
	4 or 5	N	4	238	
		%	1.7%	98.3%	
CTAS level in the second visit	1 or 2	N	7	15	**< 0.001**
		%	31.8%	68.2%	
	3	N	21	405	
		%	4.9%	95.1%	
	4 or 5	N	1	123	
		%	0.8%	99.2%	
Dementia	No	N	27	539	**0.023**
		%	4.80%	95.20%	
	Yes	N	2	3	
		%	40.00%	60.00%	
Functional dependency	Dependent	N	4	7	**0.001**
		%	36.40%	63.60%	
	Independent	N	25	535	
		%	4.50%	95.50%	
Shift period in the first visit	Morning	N	6	187	0.31
		%	3.1%	96.9%	
	Evening	N	13	197	
		%	6.2%	93.8%	
	Night	N	10	159	
		%	5.9%	94.1%	
Shift period in the second visit	Morning	N	10	208	0.825
		%	4.60%	95.40%	
	Evening	N	12	194	
		%	5.80%	94.20%	
	Night	N	7	141	
		%	4.70%	95.30%	
New to the institution	No	N	27	446	0.128
		%	5.70%	94.30%	
	Yes	N	2	97	
		%	2.00%	98.00%	
Reason for the revisit	Physician‐ or system‐related	N	3	1	**< 0.001**
		%	75.0%	25.0%	
	Disease progression	N	25	527	
		%	4.5%	95.5%	
	Adverse medication and medical intervention effects	N	1	15	
		%	6.3%	93.8%	

*Note:* Bold values indicate statistically significant associations (*p* < 0.05).

The CTAS level in the second visit, reason for the revisit, and dementia were included in the logistic regression. Simple logistic regression was performed, and the crude odds ratios (ORs) were reported. Furthermore, multiple logistic regressions with the adjusted ORs were reported (Table 3). Multiple logistic regression results revealed that the factors associated with disposition were CTAS level in the second visit, reason for the revisit, and dementia. Participants who had CTAS Level 3 had lower odds of being admitted, discharged against medical advice, or death than those who were scored CTAS Level 1 or 2 (OR, 0.128; 95% confidence interval [CI], 0.044–0.374; *p*  <  0.001).

**Table 3:** Simple and multiple logistic regression for disposition‐related factors in the second visit.

	Crude OR	Adjusted OR		95% CI for OR	*P*‐value
*CTAS level in the second visit*					
1 or 2	1	1			
3	0.111	0.128	0.044	0.374	< 0.001
4 or 5	0.017	0.015	0.001	0.169	0.001

*Reason for the revisit*					
Physician‐ or system‐related	1	1			
Disease progression	0.016	0.009	0.001	0.160	0.001
Adverse medication and medical intervention effects	0.022	0.011	0.000	0.366	0.012

*Dementia*					
Dementia (no)	1	1.000			
Dementia (yes)	13.31	9.794	1.345	71.302	0.024

Participants who had CTAS Level 4 or 5 showed lower odds of being admitted, discharged against medical advice, or death than those who were scored CTAS Level 3 (OR, 0.015; 95% CI, 0.001–0.169; *p* = 0.001).

Participants who revisited the hospital owing to disease progression exhibited lower odds of being admitted, discharged against medical advice, or death than those who revisited owing to physician‐ or system‐related causes (OR, 0.009; 95% CI, 0.001–0.160; *p* = 0.001).

Participants who revisited the hospital owing to adverse medication and medical intervention effects showed higher odds of being admitted, discharged against medical advice, or death than those who revisited owing to disease progression (OR, 0.011; 95% CI, 0––0.360; *p* = 0.012).

Participants who had dementia had higher odds of being admitted, discharged against medical advice, or death than those without dementia (OR, 9.794; 95% CI, 1.345–71.302; *p* = 0.024).

## 4. Discussion

This is a cross‐sectional study conducted in a single‐center tertiary referral hospital in Riyadh, Saudi Arabia. Our study aimed to determine the frequency of ED revisits within 72 h following discharge and assess the factors causing early ED revisits, including patient‐ and physician‐related factors.

Our study concluded that some factors were significantly associated with ED revisits within 72 h, including the patient’s CTAS level, a diagnosis of dementia, functional dependency, and the reason for the revisit. For most of the patients included in this study, comprising approximately 552 participants, the main reason for ED revisits was disease progression; of the remaining 20 patients, 16 revisits were due to adverse medication and medical intervention effects; in four patients, the revisit was due to physician‐ or system‐related causes. Several studies have indicated that the main causes of patients returning to the ED following discharge were illness‐, physician‐, and patient‐related. A prospective observational study conducted at a tertiary care center from July 2015 to June 2017 that included patients presenting to the ED within 72 h after their first visit reported that 56%, 26%, and 18% of the patients returned owing to illness‐, physician‐, and patient‐related reasons [19, 20].

Furthermore, a retrospective observational study conducted at King Fahd University Hospital in Saudi Arabia from January to May 2016 concluded that the most attributed factors for ED revisit were patient‐, physician‐, and system‐related factors [21].

Moreover, a retrospective observational study conducted at Thammasat University Hospital in Thailand from 2009 to 2010 reported that the main factors for emergency revisit within 48 h were illness‐, patient‐, physician‐, and healthcare‐related factors [22].

In this study, the CTAS, which is an ED triage algorithm that has been developed in Canada and categorizes patients into five categories according to the severity of the presenting problem, has shown an acceptable level of overall reliability in categorizing patients in the ED [23–28]. As previously mentioned, the CTAS levels in the first and second visits were statistically significant for the patient’s second disposition. The majority of patients revisiting the ED within 72 h had CTAS Level 3. A previous study has shown that most ED revisits were in Levels 4 (59.8%), 5 (36.7%), and 3 (3.3%), respectively [21]. The admission rates in the second visit were higher in CTAS Levels 1 and 2, wherein 31.8% of the participants required admission, whereas in CTAS Level 3, only 4.9% of the participants required admission on the second visit. Furthermore, the percentage of discharges in the participants with CTAS Levels 1 and 2 was higher than that of admission, discharge against medical advice, or death (31.8%). Similarly, of the participants, 95.1% were discharged at CTAS Level 3, whereas 99.2% were discharged at CTAS Level 4 or 5.

Moreover, in this study, dementia was statistically significantly associated with the type of disposition. Of the patients without dementia, 95.2% were discharged and 4.8% were admitted, died, or discharged against medical advice, whereas 60% of those with dementia were uneventfully discharged. Other ED research studies conducted in Taiwan have revealed similar findings, indicating that patients with dementia, on average, had higher admission rates and longer hospital stays [29]. LaMantia et al. also highlighted that patients who were diagnosed with dementia at the time of admission were being admitted at higher rates than patients without a current dementia diagnosis [30]. This finding is consistent with a large cross‐sectional study conducted in eight European countries between November 2010 and April 2012, which reported that hospital admission is frequent among individuals with dementia, especially among those living in the community, and imposes a significant economic burden [31].

Along with high admission rates, Jelinski et al. found dementia to be strongly associated with repeated ED revisits, and their meta‐analysis and systematic review of patients with dementia in older age reported a pooled rate of 30‐day ED revisit of 28.6%, with significant heterogeneity between studies and sites. This finding highlights that dementia is a strong risk factor for frequent ED use [32].

Furthermore, in our study, functional dependency was markedly associated with discharge disposition, wherein only 63.6% of dependent patients were discharged compared with independent patients, of whom 95.5% were discharged. This finding is consistent with a study conducted in the state of Maryland, wherein functional status on discharge, particularly the motor aspect, was strongly associated with readmission to acute care, and the functional independence measure scale predicted a need for admission [33]. Similarly, Hass et al. showed that among older adults living in the community, unmet needs for assistance with everyday living activities predicted higher ED utilization [34].

Although our study time frame (72 h) is shorter than a number of prior articles (30 days) on dementia and functional dependency [32], the high revisit burden in both populations over longer time frames supports that they represent a robust vulnerability factor for acute care use shortly after discharge and are also relevant in the very short term. Also, contextual factors related to our healthcare settings may have influenced the association between dementia, functional dependency, and higher rates of ED revisits observed in our study. Such patients have limited access to comprehensive post–discharge follow‐up, which leads to a higher burden on their care provider and a greater dependence on emergency services to manage unmet medical or assistance needs. Highlighting the importance of targeted discharge planning and post–ED support for demented and functionally dependent patients aligns with the findings of other studies in which the importance of coordinated discharge planning is emphasized in patients with dementia or significant functional dependency [32, 35].

Interestingly, our study did not demonstrate any association between having a chronic illness and the rate of emergency visits within 72 h. In this study, 306 patients (> 50%) had no comorbidities, whereas 100 and 100 patients had diabetes mellitus and hypertension, respectively. Several studies have shown that having an underlying chronic medical condition is significantly associated with an increased rate of ED revisits within 72 h and increased hospital admissions [19, 20, 36, 37]. For example, a local study conducted at King Abdulaziz Medical City, Riyadh, between September 13, 2015, and July 29, 2017, to assess 72‐h ED revisits among adults with chronic diseases concluded that the rate of 72‐h ED revisits is high among adults with chronic diseases [17]. The discrepancy between our study and the previously mentioned study could be due to the fact that their population involved adults with at least one chronic disease.

### 4.1. Limitations

This study had some limitations. First, the data presented were collected from a single center, limiting the generalizability of the findings. Second, this was a retrospective study, which is prone to missing data due to changes in documentation style over the years. Lastly, all records of revisits were independently categorized into one of the classifications on the basis of the judgment of three authors, and an inconsistent classification was noted in 20 files. These files were reviewed by the research supervisor and reclassified.

## 5. Conclusion

In summary, most patients who revisit the ED within 72 h do so due to disease progression rather than system‐ or physician‐related issues, and a substantial proportion are safely discharged on their second visit, emphasizing the need for more accessible and structured follow‐up pathways to reduce unnecessary returns. The key predictors of early ED revisits—CTAS triage level, dementia, and functional dependency—should prompt emergency physicians to implement targeted discharge planning and multidisciplinary support for vulnerable populations, particularly those with cognitive or functional impairments. Differences observed between local and international revisit patterns may reflect unique aspects of the regional healthcare system, including limitations in outpatient resources and community‐based care, highlighting the importance of tailored interventions in Saudi Arabian settings.

Practical recommendations for clinicians include proactive identification of high‐risk patients during the initial ED visit and development of care coordination strategies that integrate outpatient follow‐up, patient education, and robust discharge instructions. For healthcare systems, resource allocation toward post–discharge support—particularly for patients with dementia and functional dependency—may reduce revisit rates and improve overall care quality. Future studies should consider subclassifying disease progression and examining longitudinal outcomes to refine predictive models and support best practices in emergency medicine globally.

## Conflicts of Interest

The authors declare no conflicts of interest.

## Funding

No funding was obtained for this manuscript.

## Data Availability

The data that support the findings of this study are available from the corresponding author upon reasonable request.
